# Elbow hemiarthroplasty versus open reduction and internal fixation for AO/OTA type 13 C2 and C3 fractures of distal humerus in patients aged 50 years or above: a randomized controlled trial

**DOI:** 10.1186/s13063-020-04418-8

**Published:** 2020-06-08

**Authors:** Ali Al-Hamdani, Jeppe V. Rasmussen, Kenneth Holtz, Bo S. Olsen

**Affiliations:** Department of Orthopaedic Surgery, Herlev and Gentofte Hospital, University of Copenhagen, Copenhagen, Denmark

**Keywords:** Elbow, Hemiarthroplasty, Osteosynthesis, Humerus, Fracture, Randomized, Outcome, Reoperation, Complication

## Abstract

**Background:**

Intraarticular distal humeral fractures of AO/OTA type 13 C2 and C3 pose a surgical challenge despite the evolution of surgical implants and techniques. Open reduction and internal fixation (ORIF) is often preferred as the first choice of treatment, but the results vary and are sometimes disappointing. Total elbow arthroplasty (TEA) has been widely used for fractures that are not amenable to ORIF in elderly patients, but the mechanical complications remain a challenge, especially in active patients. Elbow hemiarthroplasty (EHA) provides a modern alternative that might avoid the mechanical complications and weight bearing restrictions related to the linked articulation in semi-constrained TEA. No studies have compared the results of EHA to that of ORIF, but case series have reported promising results.

**Methods/design:**

This is a study protocol describing an investigator-initiated, non-blinded randomized controlled trial comparing the outcome of EHA with ORIF for AO/OTA type 13 C2 and C3 fractures of the distal humerus in patients who are 50 years or older. Forty-four patients with AO/OTA type 13 C2 and C3 fractures of distal humerus will be randomized to either EHA or ORIF. The Oxford Elbow Score (OES) will be used as primary outcome. Mayo Elbow Performance Score (MEPS), pain severity score (VAS), range of motion, and patient satisfaction will be used as secondary outcomes. Reoperations, complications, and the length of sick leave will be recorded. The patients will be examined after the operation and at 3 months and 1, 2, 5, and 10 years.

**Discussion:**

The main objective of this study is to investigate the best treatment option for AO/OTA type 13 C2 and C3 fractures of distal humerus in patients aged 50 years or above. We hypothesize that EHA results in fewer complications and superior functional outcome compared with ORIF and that the mechanical complications related to the linked articulation of TEA can be avoided.

**Trial registration:**

ClinicalTrials.gov, PRS, NCT04163172. Registered November 13, 2019. https://clinicaltrials.gov/ct2/results?cond=&term=evori&cntry=&state=&city=&dist= (Table 2).

The protocol has been approved by The Scientific Ethics Committee of the Capital Region of Denmark (Jr. no.: H^− 19,035,590^).

The processing of personal data has been approved by the Danish Data Protection Agency (Jr. no. P-2019-246). Inclusion started on February 1, 2020.

## Background

Adult distal humeral fractures comprise 2% to 5% of all fractures and 30% of all elbow fractures [2, 22]. Despite the evolution of surgical implants and techniques, AO/OTA type 13 C2 and C3 fractures of the distal humerus still pose a surgical challenge. Open reduction and internal fixation (ORIF) is often preferred as the first choice of treatment, but the results vary and are sometimes disappointing, especially in elderly patients [10, 25, 29]. The success of ORIF depends on correct reduction of the fracture, reconstruction of the articular surface, and the stability and rigidity of the fixation [12]. Total elbow arthroplasty (TEA) has been widely used during the last 20 years for fractures in elderly patients that are not amenable to ORIF, but the mechanical complications related to the linked articulation remain a challenge [7, 23, 27]. Unlinked TEA has been used to minimize the mechanical complications of the linked TEA, but the joint instability is a major complication, especially in condylar displacement, and the survival rate and functional results may be lower than for the linked arthroplasty [14, 16, 30, 31]. Elbow hemiarthroplasty (EHA) provides a modern alternative that might avoid the mechanical complications and weight bearing restriction related to the linked articulation in TEA [1, 19, 20, 24, 26].

Currently, EHA has not been compared to ORIF, but previous studies comparing the outcomes of TEA and ORIF in elderly patients with an intraarticular distal humeral fracture concluded that good outcomes may be obtained with both procedures, but TEA resulted in more predictable outcome with fewer reoperations and major complications [8, 9, 11, 17].

The objective of this study protocol is to describe the methodology for an investigator-initiated, randomized controlled trial comparing the outcomes of EHA and ORIF for AO/OTA type 13 C2 and C3 fractures of distal humerus in patients who are 50 years or older. The Standard Protocol Items Recommendations for Interventional Trials (SPIRIT) Statement 2013 has been followed for the completion of this protocol (Table 1 and Additional file 1).

**Table 1:**
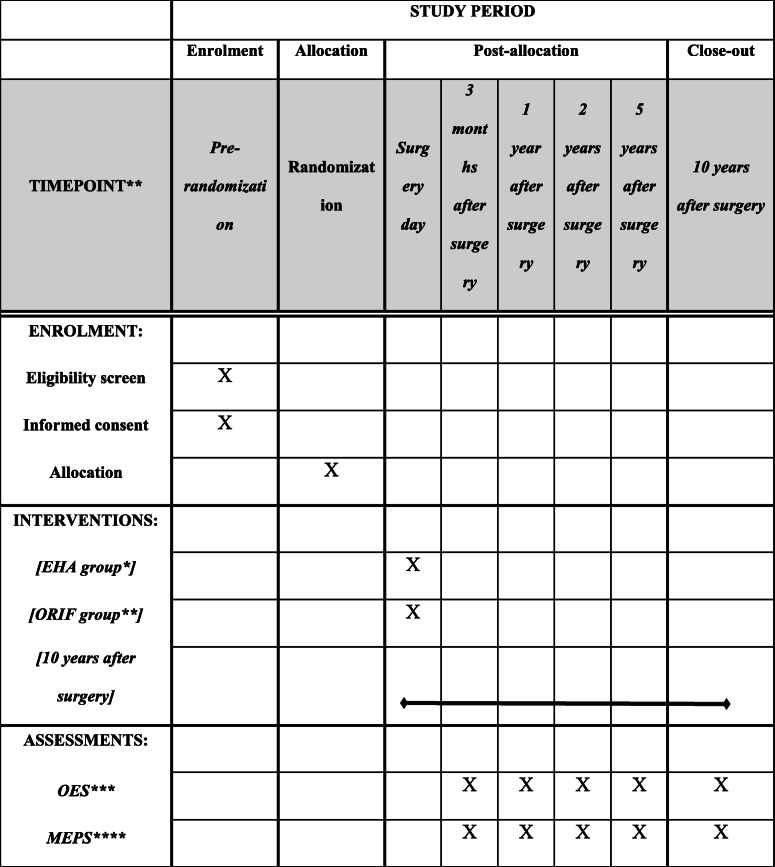
Standard Protocol Items Recommendations for Interventional Trials diagram (SPIRIT)

* EHA group operated with Latitude anatomical hemiarthroplasty (WRIGHT, Memphis, TN, USA)

** ORIF group operated with double plates (Synthes, Switzerland and West Chester, PA, US)

***OES Oxford Elbow Score

****MEPS Mayo Elbow Performance Score

## Methods/design

### Study design and aim

This is an investigator-initiated randomized controlled trial. The main objective of the study is to compare cemented hemiarthroplasty with open reduction and internal fixation of AO/OTA type 13 C2 and C3 fractures of the distal humerus in patients aged 50 years or above.

### Subjects

Forty-four patients with AO/OTA type 13 C2 and C3 fractures of the distal humerus randomized to either EHA or ORIF.

### Inclusion criteria

1. AO/OTA type 13 C2 and C3 fractures of the distal humerus confirmed by plain radiographs with two perpendicular views and CT scan
2. ASA score 1–3 and physically fit for surgery
3. Age of 50 years or above

### Exclusion criteria

1. Patients unable to follow the rehabilitation protocol or answer the Danish questionnaires because of physical or cognitive inabilities as evaluated by the recruiting surgeon
2. Significant elbow osteoarthritis as evaluated by the recruiting surgeon based on plain radiographs and CT scan
3. Fractures that are older than 6 weeks
4. Other associated elbow fractures
5. Pathological fractures or relevant elbow pathology

### Screening and data collection preoperatively

1. Gender and age
2. Plain radiographs with standard anterior-posterior and lateral projections
3. CT scan of the injured elbow joint

### Eligibility

All Danish citizens aged 50 years or above with AO/OTA type 13 C2 and C3 fractures of the distal humerus referred to the orthopedic department at Herlev and Gentofte University Hospital will be offered participation in the trial. The patients will receive oral and written information about the study by the operating surgeon or by the primary investigator. They will have at least 24 h to consider participation in the study before informed consent is obtained. They will also be informed that they can withdraw their consent at any time. The informed consent gives the primary investigator access to information about the patient’s health condition from the medical record. The study will follow the Helsinki Declaration.

All the operations will be performed at the orthopedic department at Herlev and Gentofte University Hospital, which is the largest referral shoulder and elbow unit in Denmark. Fifteen to twenty patients with this type of fracture have been treated at the department each year from 2016 to 2018. With this number of patients, we estimate that a 3-year recruitment period from February 1, 2020 to December 31, 2022 will allow inclusion of the planned number of patients.

### Randomization and concealment of allocation

Based on the sample calculation, we intend to include a total number of 44 patients allocated into two groups of equal size:

1. EHA (elbow hemiarthroplasty)—intervention group
2. ORIF (open reduction and internal fixation)—comparator group

The randomization is done just prior to the operation by the primary investigator, who will inform the operation team about the intended surgery. The randomization sequence will be computer generated using RED-Cap (Research Electronic Data Capture) program. The patients will be randomized using stratified, block randomization with a 1:1 allocation ratio and stratified according to age in years (50–69, 70 and above) and sex. The randomization sequence is generated by a statistician, and the patients will be stratified and then randomized. After randomization, the patient’s allocation will be revealed to the operation team to unpack the necessary operation tools and prosthesis.

### Blinding

This randomized controlled trial is not blinded to patients, theater staff, surgeons, physiotherapists, or the principle investigator. The secondary outcomes are evaluated, unblinded, by the primary investigator. The primary outcome, the Oxford Shoulder Score, is a survey and data collection does not involve surgeons, the primary investigator, or any other persons affiliated with the study. The data analysis will be blinded.

All Danish citizens have access to their own medical record and x-rays through an internet-based system which makes blinding laborious and expensive. Furthermore, the patients can probably feel the plates beneath the skin in case of ORIF, which makes it impossible to ensure that the patients remain blinded during the study period.

### Interventions

All patients will be operated on by one of three experienced elbow surgeons in order to secure a high surgical standard. The treatment is standardized; all patients have a tourniquet and are placed in a lateral supine position. A posterior midline approach will be used, and the ulnar nerve will be identified and protected in situ without transposition. All procedures are performed using a triceps-split.

#### The Latitude anatomical hemiarthroplasty (WRIGHT, Memphis, TN, US)

The prosthesis will be used according to the manufacturer’s manual. A trial component will be inserted, and range of motion and stability will be evaluated before the component is inserted using a standard cementing technique. The medial and lateral epicondyles, with the collateral ligaments attached, will be fixated to the arthroplasty using this FiberWire suture (Arthrex, Naples, FL, USA) and subsequently to the humerus using additional FiberWire sutures. In patients with a preserved medial column, the medial ligament will not be detached during the procedure.

#### Double plating (Synthes, Switzerland and West Chester, PA, US)

The fracture will be reduced and the articular surface will be reconstructed as well as possible. The fracture will then be fixated with two plates—parallel or perpendicular according to the fracture nature. The collateral ligaments will be attached to the plates using FiberWire suture (Arthrex, Naples, FL, USA). The stability and rigidity of the fixation will be checked.

Standard closure with absorbable sutures and reinsertion of the collateral ligaments with non-absorbable sutures will be performed. Standard skin closure is with with metal clamps. All patients will receive standard pre-, peri-, and post-operative pain management, including general anesthesia and interscalene peripheral nerve block performed by the anesthesiologist. The pain management can be adjusted and individualized if needed. There is no collection and storage of biological specimens for genetic or molecular analysis.

### Physiotherapy and rehabilitation

A back splint will be used for 3 to 4 days. The patients will perform edema prophylactic exercises during the time of immobilization. All patients will follow a standard rehabilitation program supervised by a physiotherapist. This includes active flexion and passive extension of the elbow the first 2 weeks, active flexion and extension without any weight bearing from 3 to 6 weeks, and weight bearing after 6 weeks. Full weight-bearing exercises are allowed after 3 months.

### Outcome measures

#### Primary functional outcome

The Oxford Elbow Score (OES) will be used to assess the primary outcome at 2 years.

The OES is a 12-item, patient-administrated questionnaire that measures the quality of life in patients with an elbow disorder. There are three unidimensional domains: elbow function, pain, and social-psychological status [5, 13]. Each question is answered on a five-point scale with each question contributing equally to the total score. Thus, the total score ranges from 12 to 60, with 60 being the worst. For ease of presentation the score is converted to a scale from 0 to 48 with 48 being the best. The outcome can be interpreted based on a 48-point scale: 0–19, poor; 20–29, fair; 30–39, good; and 40–48, excellent [6]. The Danish version, which will be used in this study, has been translated and culturally adapted according to the guidelines by Guillemin, Bombardier, and Beaton [6, 21].

The minimal clinically important differences (MCID) for the total score of OES has not been defined, but Dawson et al. reported the MCID for each of the three domains, pain, function, and social-psychological. The MCID for the three domains was 19, 9, and 18, respectively [5]. Accordingly, we estimated the MCID for the total score to be 15.

#### Secondary functional outcomes

##### Mayo Elbow Performance Score

Mayo Elbow Performance Score (MEPS) is a surgeon-administrated instrument that evaluates the outcome after elbow surgery [18]. There are four domains, including pain (0–45 points), range of motion (0–20 points), stability (0–10 points), and difficulties in daily activities (0–25 points) [3, 4]. The outcome can be interpreted based on a 100-point scale: 0–60, poor; 60–74, fair; 75–89, good; and 90–100, excellent [18]. The MCID has been defined as 10 [4].

##### Pain severity score

Pain is answered on a visual analogue scale (VAS) ranging from 0 to 10 (on a 100-mm scale), with 10 being the worst and 0 representing a pain-free elbow [15]. The MCID has been defined as 20 mm (two points) [28].

##### Range of motion

Range of motion (ROM) will be measured in degrees, both flexion/extension arc and supination/pronation arc. To our knowledge, the MCID of ROM in patients with distal humeral fractures has never been reported.

##### Patient satisfaction

Patient satisfaction of the treatment will be recorded using a five-item score (5, very satisfied; 4, satisfied; 3, neither satisfied nor dissatisfied; 2, dissatisfied; and 1, very dissatisfied).

#### Radiographic evaluation

Radiographic evaluation will be performed using anteroposterior and lateral radiographs. There is no specific and validated method to evaluate x-ray images after EHA/ORIF or to evaluate post-traumatic osteoarthritis of the elbow joint, so decreased height of the humeral, ulnar, and radial cartilage, bone healing and joint alignment, arthroplasty/plates loosening, and the presence of heterotopic ossification will be analyzed as binary outcomes (yes or no) by the primary investigator.

#### Complications and reoperations

We will record all complications related to the surgical procedure (nerve injuries, deep and superficial infections, malpositioning of the components, and instability) and reoperations (defined as any surgical intervention to the index elbow after the primary procedure).

Length of sick leave will be recorded.

### Follow-up

All patients will be followed actively for 10 years. To ensure the implant position, all patients will undergo a plain x-ray in two planes before discharge, which will usually be done on the first postoperative day. Then all patients will be followed actively with plain radiographs, clinical outcome, and patient-reported outcome at 3 months postoperatively and at 1, 2, 5, and 10 years.

### Protocol violations, patient drop-out, and revision

The number of patients who receive the allocated treatment will be reported. If ORIF is technically impossible, the surgeon remains free to use an EHA if it is believed to be in the best interest of the patient. A main analysis will be conducted on an intention-to-treat basis where all outcome scores from each patient are analyzed according to the randomization, irrespective of type of surgery received. If operative crossover does occur, a secondary analysis will be conducted on a per protocol basis. This analysis will only include patients who undergo surgery according to the randomization.

Patients who drop out of the trial will be recorded, and the reason for the drop-out will be noted. The patient will be included in the analysis with the latest follow up (last observation carried forward). Drop-outs before the 3-month evaluation cannot be included in the analysis due to lack of preoperative data. The number of patients who do not comply with rehabilitation will be recorded.

If a reoperation is needed, the reason and the operation type are recorded. If possible, the patient is evaluated using clinical outcome and patient-reported outcome prior to the revision procedure. The patient will remain in the study and they are included in the analysis with their latest follow-up.

### Side effects and adverse event reporting

Both types of treatment have been used routinely in our department and specifically by all participating surgeons. The treatments’ risks and disadvantages are therefore not expected to differ from normal patient application outside the trial. All expected and unexpected complications related to the surgical procedure (nerve injuries, deep and superficial infections, malpositioning of the components, and instability) will be recorded systematically by the primary investigator. The patients are routinely asked about complications at each follow-up. Information about hospital contacts related to the surgical procedure is also retrieved from the institutional medical record and from the Danish National Patient Register, which is a nationwide database recording all hospital contacts in Denmark.

Both ORIF and EHA have proven safe in retrospective series from our institution and the study will not be stopped prematurely.

### Statistics

#### Sample size calculation OES

Oxford elbow score (OES) will be used to measure the primary outcome.

Sample size calculation was performed with a standard deviation (SD) of 16, a MCID of 15, a significance level at 0.005, and power of 0.80, resulting in 18 participants in each group (44 participants in all with allowance for 18% drop-out).

The estimation of SD = 16 is extrapolated from a study on 24 patients with distal humeral fractures treated with EHA [1]. There is no MCID defined for the total score of OES, but Dawson et al. have reported the MCID for each of the three domains—pain, function, and social-psychological—for a group of patients with different pathologies including TEA for osteoarthritis and rheumatoid arthritis. MCID for the three domains is 19, 9, and 18, respectively [5]. Accordingly, we chose the MCID to be 15.

#### Hypotheses

We hypothesize that at 2 years, EHA provides a superior OES compared with ORIF by at least a difference of 15.

#### Data analysis

No interim data analysis will be carried out before the analysis at 2 years. Baseline characteristics and outcome scores will, for each group, be summarized by number and percentage (categorical data) and mean and standard deviation or median and interquartile range, as appropriate (continues data). No statistical tests of differences in baseline characteristics between the two groups will be conducted as any difference must be due to chance and not bias. The differences in outcome scores between the two groups will be analyzed with use of parametric statistics (Students *t*-test) or non-parametric statistics (Mann-Withney U-test) as appropriate. Categorical data will be analyzed with use of chi-square test.

## Discussion

We will attempt to avoid the type-II error (when the hypothesis testing does not reject the null hypothesis, even though the null should have been rejected) by using a proper sample size and by adding 18% to sample size compensating for drop-outs. The drop-out bias will be minimized by obtaining permission to call patients who do not show up for subsequent follow-up and also to visit the patients at home. Selection bias will be avoided through randomization. If ORIF during the surgery is regarded to be technically impossible, the procedure can be converted to EHA. In order to follow the principle of the intention-to-treat, the patient will continue to be in the ORIF group and included in the analysis according to the randomization. A secondary analysis will be conducted on a per protocol basis where only patients who undergo surgery according to the randomization are included.

The consequences of having unblinded patients are unknown. It may introduce bias to the study, which, in most cases, will be in favor of the intervention. In the present study, the intervention and the comparator are surgery and the patients and blinding of the patients may not be as important as in studies where surgery is compared with non-surgery. The primary outcome is a patient-administered questionnaire and does not involve any persons affiliated with the study. It was chosen as the primary outcome to avoid observer bias. The secondary outcomes are not observer blinded and the results will be interpreted carefully due to the risk of bias.

The trial will provide high-quality evidence regarding the short- and long-term clinical and patient-reported outcomes of ORIF and EHA. We use the same outcome scores as in previous randomized trials comparing TEA and ORIF [8, 9, 17]. This makes it possible not only to compare the results but also to include the results in future systematic reviews and meta-analyses. We believe that the results from the present trial, together with results from other randomized trials, can be used to establish a treatment algorithm for distal, intraarticular humerus fractures.

### Trial status

Protocol version 4.

Issue date: 28/04/2020, author Ali Al-Hamdani.

Inclusion started on February 1, 2020. Finnish date of recruitment of all patients is expected to be at December 31, 2022. In case the target number of 44 patients has not been met, the recruitment period may be extended to reach the number required. The trial processes will be monitored by the primary investigator and the study group to ensure the protocol. If any changes are necessary, the primary investigator is obliged to inform The Scientific Ethical Committee of the Capital Region of Denmark and Data Protection Agency.

## Supplementary information

**Additional file 1.** SPIRIT 2013 Checklist: Recommended items to address in a clinical trial protocol and related documents.

## Data Availability

All study-related information will be stored securely on the study site, including all completed paper forms. Study data will be collected and managed using REDCap electronic data capture tools and EPIC hospital clinical digital journal system hosted in the Capital Region of Denmark. REDCap is a secure, web-based application designed to support data capture for research studies. Identifying information of the patients will be stored in the hospital’s secured intranet system. Study information will not be released without the permission of the relevant participant. The primary investigator will do the data collection and will have access to the trial materials and final dataset. The study is approved by the Danish Data Protection Agency, and it will follow General Data Protection Regulation and Data Protection Act. After the completion of the study, the results will be made available for meta-analysis and systematic reviews.

## Abbreviations

EHA: Elbow hemiarthroplasty
ORIF: Open reduction and internal fixation
TEA: Total elbow arthroplasty
OES: Oxford Elbow Score
MEPS: Mayo Elbow Performance Score
VAS: Pain severity score
CT: Computerized tomography
RED-Cap: Research Electronic Data Capture
MCID: Minimal clinically important differences
ASA: American Society of Anesthesiologists Score
AO/OTA: Arbeitsgemeinschaft für Osteosynthesefragen/Orthopaedic Trauma Association
